# Supplementary material for: Machine Learning Reveals Signatures of Promiscuous Microbial Amidases for Micropollutant Biotransformations
Source: ACS Environ Au. 2024 Dec 4;5(1):114–27. doi: 10.1021/acsenvironau.4c00066 (PMC11741061; doi:10.1021/acsenvironau.4c00066)
Supplement: Supplementary file 1 — vg4c00066_si_001.pdf [file vg4c00066_si_001.pdf]

Supporting Information for:

Machine learning reveals signatures of promiscuous microbial amidases for micropollutant biotransformations

Thierry D. Marti [a,b], Diana Schweizer [a,c], Yaochun Yu [a], Milo R. Schärer [a,d], Silke I. Probst [a], Serina L. Robinson [a*]

Author affiliations:

[a] Eawag, Swiss Federal Institute of Aquatic Science and Technology, 8600 Dübendorf, Switzerland

[b] Institute of Biogeochemistry and Pollutant Dynamics, ETH Zürich, 8092 Zürich, Switzerland

[c] Institute for Ecopreneurship, University of Applied Sciences and Arts Northwestern Switzerland, 4132 Muttenz, Switzerland

[d] Institute of Food, Nutrition and Health, ETH Zürich, 8092 Zürich, Switzerland

*Corresponding author: serina.robinson@eawag.ch

Supplementary Methods

Substrate specificity screening of P205 using micropollutants

Stock solutions of individual micropollutants were prepared in dimethyl sulfoxide (DMSO) at a concentration of 10 mM and stored at −20°C. These micropollutants were then divided into 14 submixtures, mixed and diluted with ethanol to achieve a final working concentration of 200 μM for each micropollutant.

Micropollutant biotransformations catalyzed by the enzyme P205 were investigated in a clear-bottomed 96-well plate (Greiner) as described previously with slight modifications.[1] Briefly, individual micropollutant sub-mixtures were first added to an empty 96-well plate. The plate was placed in a fume hood to allow the organic solvent to completely evaporate. After evaporation, 239 μL of 100 mM Tris-HCl buffer (pH = 8) was added to each well. The plate was placed on a rotary shaker at 50 rpm for 15 min to redissolve the micropollutants. Purified P205 in 11 μL aliquots were added to a 96-well plate to achieve a final enzyme concentration of ~190 nM per well and mixed thoroughly to initiate the biotransformation reactions. Samples were collected at 0 h, 6 h, and 24 h. At each sampling event, a 20 μL sample was taken and mixed with 40 μL pure methanol to completely denature the enzyme and quench the reaction, followed by the addition of 140 μL Tris-HCl buffer to the sample mixture. After centrifugation (13,000 × g, 4°C for 15 min), the supernatant was collected and stored at 4°C for subsequent measurement by ultrahigh-performance liquid chromatography coupled to high-resolution tandem mass spectrometry (UHPLC-HRMS/MS). An abiotic buffer control and inactivated enzyme (P205-S146A) control were set up in the same way as the experiment described above. UHPLC-HRMS/MS analysis was conducted as described below. Matrix match calibration standards were used for quantification. The quantification was based on concentrations calculated from peak area integration using TraceFinder 5.1 (Thermo Fisher Scientific).

UHPLC-HRMS/MS analysis

The micropollutants were measured by UHPLC-HRMS/MS (Q Exactive, Thermo Fisher Scientific) using a method as previously described.[1] Briefly, for the LC separation, 25 μL samples were injected into an ACQUITY Premier BEH C18 Column (particle size 1.7 μm, 100 × 2.1 mm, Waters) and eluted with Nanopure water (A) and methanol (B), both containing 0.1% formic acid, at a flow rate of 300 μL/min. A linear elution gradient was set as follows: 95% A: 0 – 1.5 min, 95% – 5% A:1 – 7.5 min, 5% A: 7.5 – 9.5 min, and 95% A: .5 – 11.5 min. For HRMS, mass spectra were acquired in full scan mode at a resolution of 70,000 at m/z 200 and a scan range of m/z 100 − 1000 in positive/negative switching electrospray ionization mode. Xcalibur 4.0 (Thermo Fisher Scientific) and TraceFinder 5.1 (Thermo Fisher Scientific) were used for data acquisition and analysis. Compounds exhibiting more than 40% parent compound removal by P205and negligible removal in abiotic and inactivated (P205-S146A) controls were selected for further investigation.

Supplementary Tables

Table S1: Substrates and compounds used in this study

**Compound**	**Supplier**
acesulfame	Sigma Aldrich
atenolol	Chemie Brunschwig
acetylsulfamethoxazole	Chemie Brunschwig
atorvastatin	Chemie Brunschwig
capecitabine	Chemie Brunschwig
chlorpropham	Chemie Brunschwig
diuron	Chemie Brunschwig
dasatinib	Sigma Aldrich
metoclopramide	Chemie Brunschwig
propanil	Sigma Aldrich
rufinamide	Chemie Brunschwig
vorinostat	Chemie Brunschwig
4-nitrophenyl butyrate	Sigma Aldrich
4-nitrophenyl trimethylacetate	Sigma Aldrich
flutamide	Sigma Aldrich
nitroacetanilide	Sigma Aldrich
paracetamol	Sigma Aldrich
4-nitrophenol	Sigma Aldrich
nitroaniline	Sigma Aldrich
4-nitro-3-(trifluoromethyl)aniline	Sigma Aldrich
sulfamethoxazole	Sigma Aldrich
5’-deoxy-5-fluorocytidine	Chemie Brunschwig
4-nitro-3(trifluoromethyl)aniline	Sigma Aldrich

Table S2: Solutions for protein purification using benchtop purification

**Buffer**	**Composition**
equilibration buffer	20 mM Tris-HCl, 500 mM NaCl, 10% glycerol, 20 mM imidazole, pH 8
wash buffer	20 mM Tris-HCl, 500 mM NaCl, 10% glycerol, 40 mM imidazole, pH 8
elution buffer	20 mM Tris-HCl, 500 mM NaCl, 10% glycerol, 500 mM imidazole, pH 8
SGT buffer	5 mM Tris-HCl, 30 mM NaCl, 10 % glycerol, pH 8

Table S3: Shorthand notation, NCBI accession numbers, and source organisms for the amidase signature enzymes investigated in this study.

Shorthand notation	Accession number	Source organism
P109	PLA27924.1	*Corynebacterium coyleae*
P110	PKZ65245.1	*Gordonia terrae*
P111	PLA20978.1	*Corynebacterium pyruviciproducens*
P113	PKZ43269.1	*Globicatella sanguinis*
P115	PMC16657.1	*Oligella urethralis*
P116	PLA35052.1	*Corynebacterium amycolatum*
P117	PLR73545.1	*Bacillus* sp.
P118	PKY70760.1	*Brevibacterium ravenspurgense*
P121	PLB08528.1	*Pseudomonas aeruginosa*
P122	PLA16723.1	*Klebsiella pneumoniae*
P124	PLA23573.1	*Cutibacterium acnes*
P128	PKZ01497.1	*Enterococcus faecalis*
P131	PKZ94153.1	*Streptococcus salivarius*
P140	PLA78093.1	*Streptococcus agalactiae*
P145	PKY92026.1	*Aerococcus christensenii*
P146	PLR70916.1	*Bacillus* sp.
P148	PKZ20744.1	*Aerococcus sanguinicola*
P149	PKZ65816.1	*Gordonia terrae*
P150	PLT14322.1	*Limosilactobacillus fermentum*

P151	PLB82905.1	*Actinotignum timonense*
P152	PLB04707.1	*Pseudomonas aeruginosa*
P153	PLR66227.1	*Bacillus* sp.
P154	PMC29638.1	*Lactobacillus iners*
P156	PKY89575.1	*Falseniella ignava*
P157	PLA40941.1	*Neisseria sicca*
P161	PKY92403.1	*Facklamia hominis*
P162	PKY89520.1	*Falseniella ignava*
P163	PKZ08631.1	*Dermabacter hominis*
P167	PKY70105.1	*Brevibacterium ravenspurgense*
P168	PKY79758.1	*Pseudoglutamicibacter albus*
P180	PKZ05429.1	*Enterococcus faecalis*
P182	PKZ37694.1	*Kocuria rhizophila*
P190	PKZ63448.1	*Gordonia terrae*
P192	PKY80781.1	*Pseudoglutamicibacter albus*
P197	PKY93381.1	*Facklamia hominis*
P198	PLA79535.1	*Streptococcus agalactiae*
P200	PKY92027.1	*Aerococcus christensenii*
P203	PKZ25206.1	*Corynebacterium aurimucosum*
P204	PKZ66103.1	*Gordonia terrae*
P205	PLA55254.1	*Lacticaseibacillus rhamnosus*

Table S4: Leave-one-chemical-out validation of the gradient boosting model.

Chemical omitted from model training	F1 score	Balanced accuracy
4NP-butyrate	0.50	67%
4NP-trimethylacetate	0.74	69%
acesulfame*	0.77	NA
acetylsulfamethoxazole	0.86	50%
atenolol*	0.77	NA
atorvastatin*	0.81	NA
capecitabine	0.86	50%
chlorpropham*	0.67	NA
dasatinib*	1.00	NA
diuron*	0.81	NA
flutamide	0.95	92%
metoclopramide*	1.00	NA
nitroacetanilide	0.95	95%
paracetamol	0.70	56%
propanil	0.67	50%
rufinamide*	0.81	NA
vorinostat	0.77	50%

*one-class compounds (not biotransformed by any enzyme) for which accuracy cannot be calculated.

Table S5: Leave-one-enzyme-out validation of the gradient boosting model.

Enzyme omitted from model training	F1 score	Balanced accuracy
P109	0.97	75%
P117	0.94	50%
P118	0.97	97%
P128	0.86	60%
P131	0.67	56%
P140	0.81	84%
P148	0.84	83%
P151	0.86	59%
P156	0.87	82%
P161	0.92	83%
P162	0.75	63%
P167	0.87	41%
P168	0.81	58%
P182	0.94	47%
P197	0.97	50%
P198	1.00	100%

Supplementary Figures

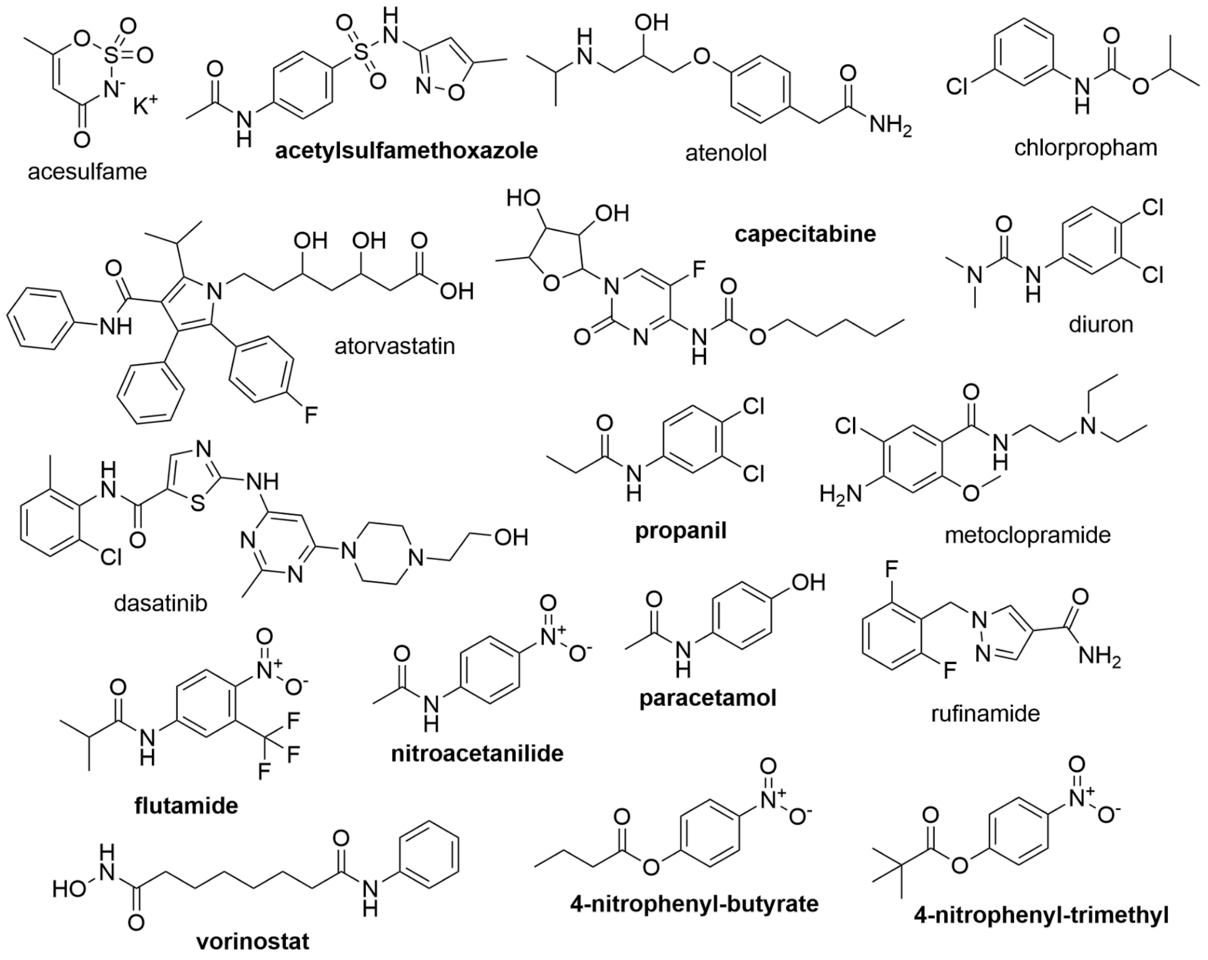

Figure S1: Chemical structures of the substrates tested in this study against the AS enzyme library. Bold names indicated substrates that have been biotransformed by at least one enzyme.

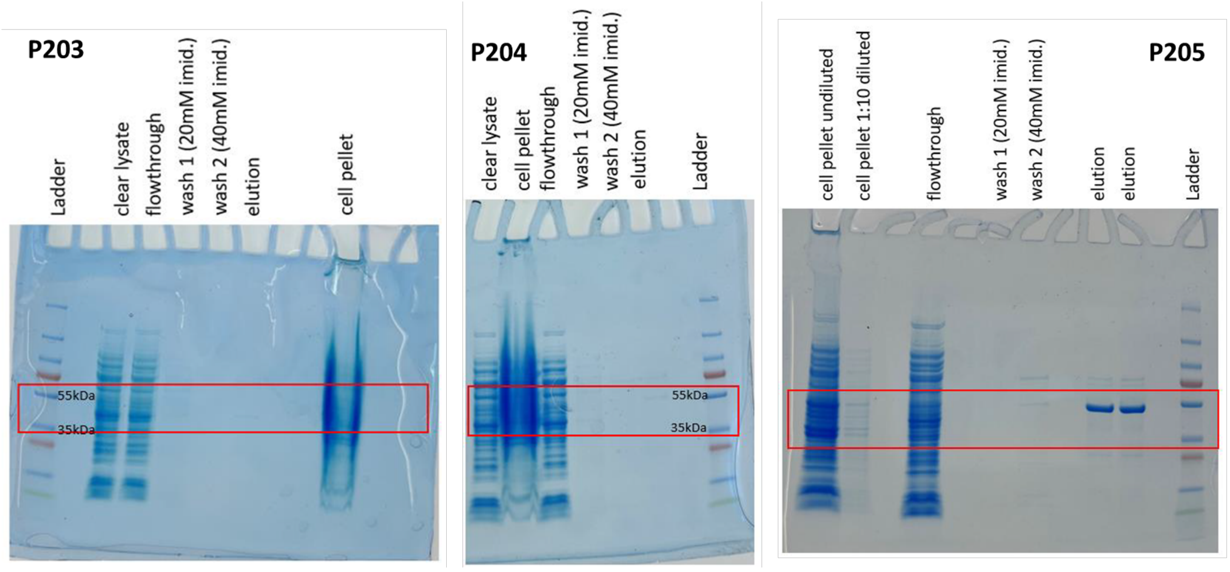

Figure S2: SDS-PAGE gels of protein purification of P203, P204 and P205 via his-tag affinity chromatography. Red boxes show protein bands between sizes of 35 to 55 kDa.

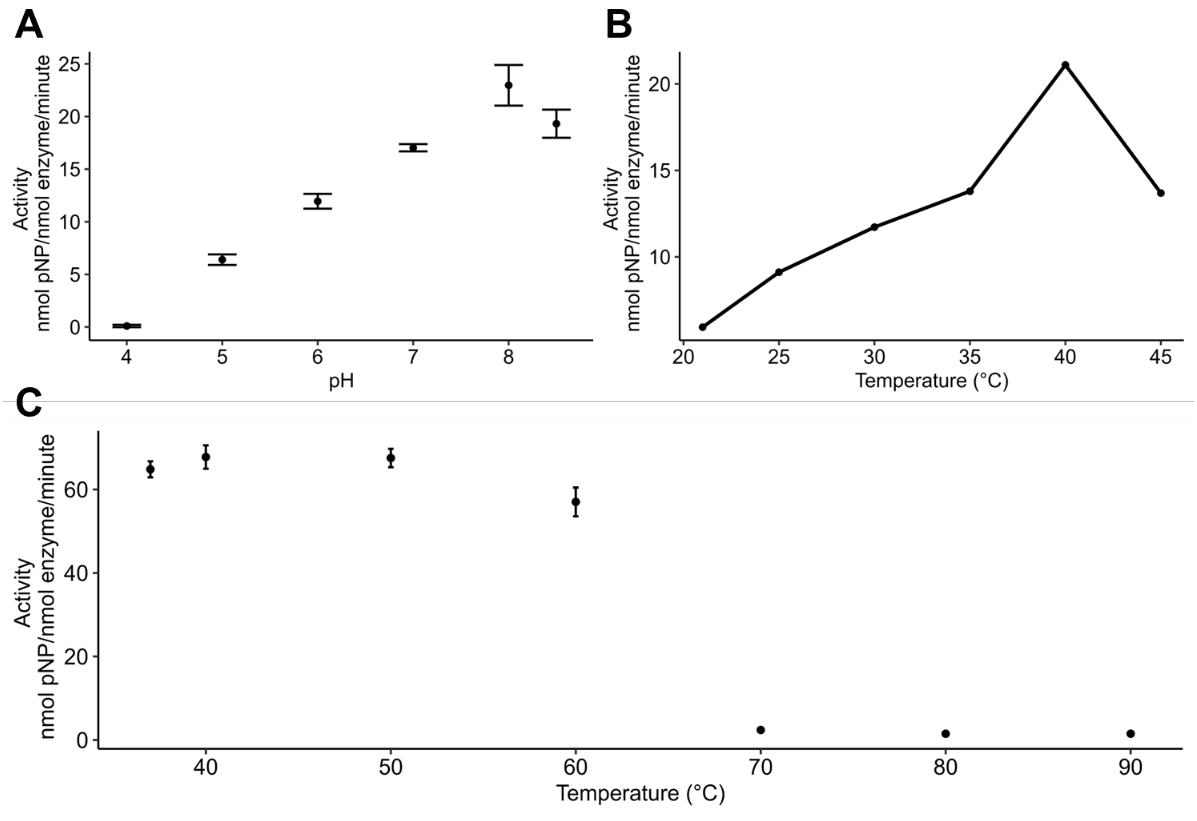

Figure S3: Biochemical characterization of P205. A) The P205 pH optimum was determined to be pH 8.0 by measuring the isosbestic point of 4-nitrophenol (347 nm) across pH 4-9 with the substrate 4-nitrophenyl trimethylacetate. Citrate buffer was used for pH 4-6 and Tris-HCl buffer was used for pH 7-9. Enzyme activity measurements for pHs higher than 9 are not shown due to rapid abiotic hydrolysis of 4-nitrophenyl trimethylacetate. B) The P205 temperature optimum was determined measuring 4-nitrophenol absorbance at 410 nm in Tris-HCl buffer (pH 8) across different temperatures using 4-nitrophenyl trimethylacetate. C) The heat stability of P205 was assessed by measuring 4-nitrophenol absorbance at 410 nm in Tris-HCl buffer (pH 8) after exposure to various temperatures for 1 hour using 4-nitrophenyl-butyrate. All experiments were conducted using purified P205 (final concentration of 0.0022 mg/mL for the temperature optimum, 0.022 mg/mL for the pH optimum and heat stability).

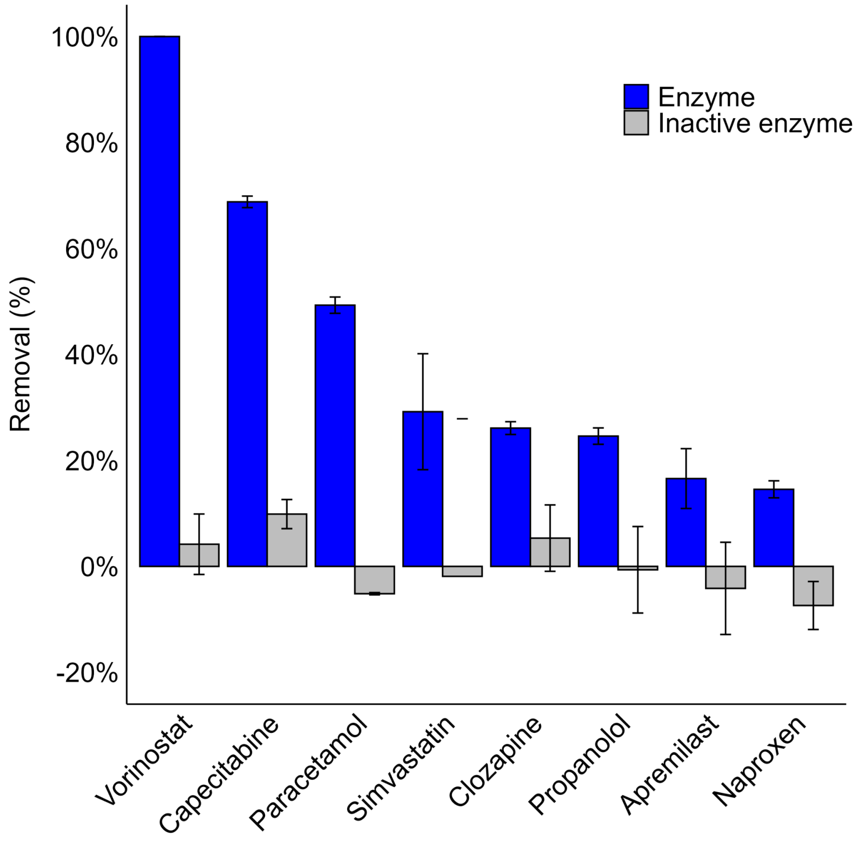

Figure S4: P205 (blue) substrate specificity screening hits (with >20% removal difference between enzyme and inactive enzyme) after 24 h incubation with a collection of a library of 183 micropollutants. The inactive enzyme control (gray) is the catalytically inactive variant P205-S146A.

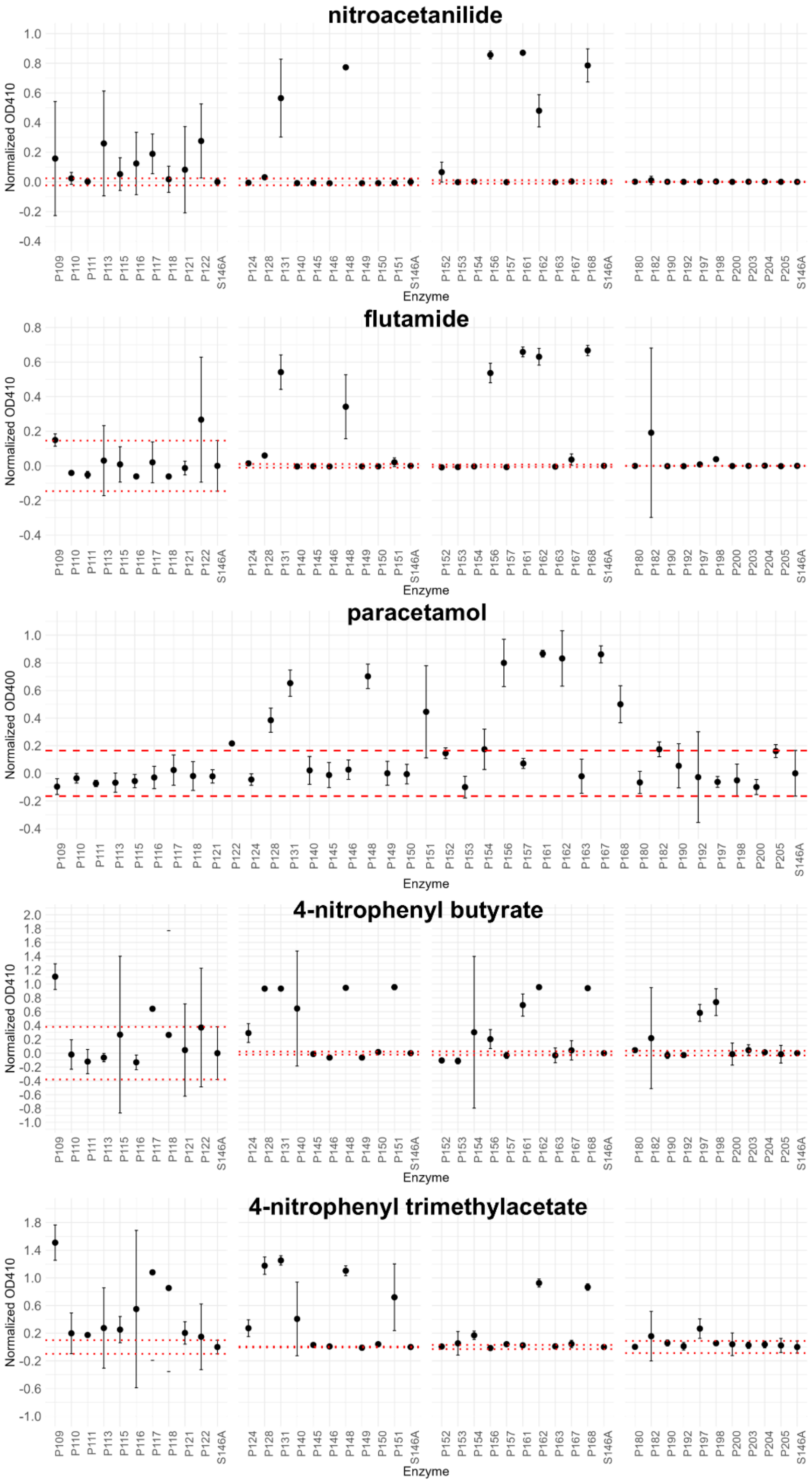

Figure S5: Endpoint OD measuring chromophores (see Methods) produced by the activity of the AS enzyme library. The OD was normalized by subtracting the OD of the inactivated enzyme control (P205-S146A, depicted as S146A). The red dotted lines represent two standard deviations from the mean activity of P205-S146A. Data is based of biological triplicates.

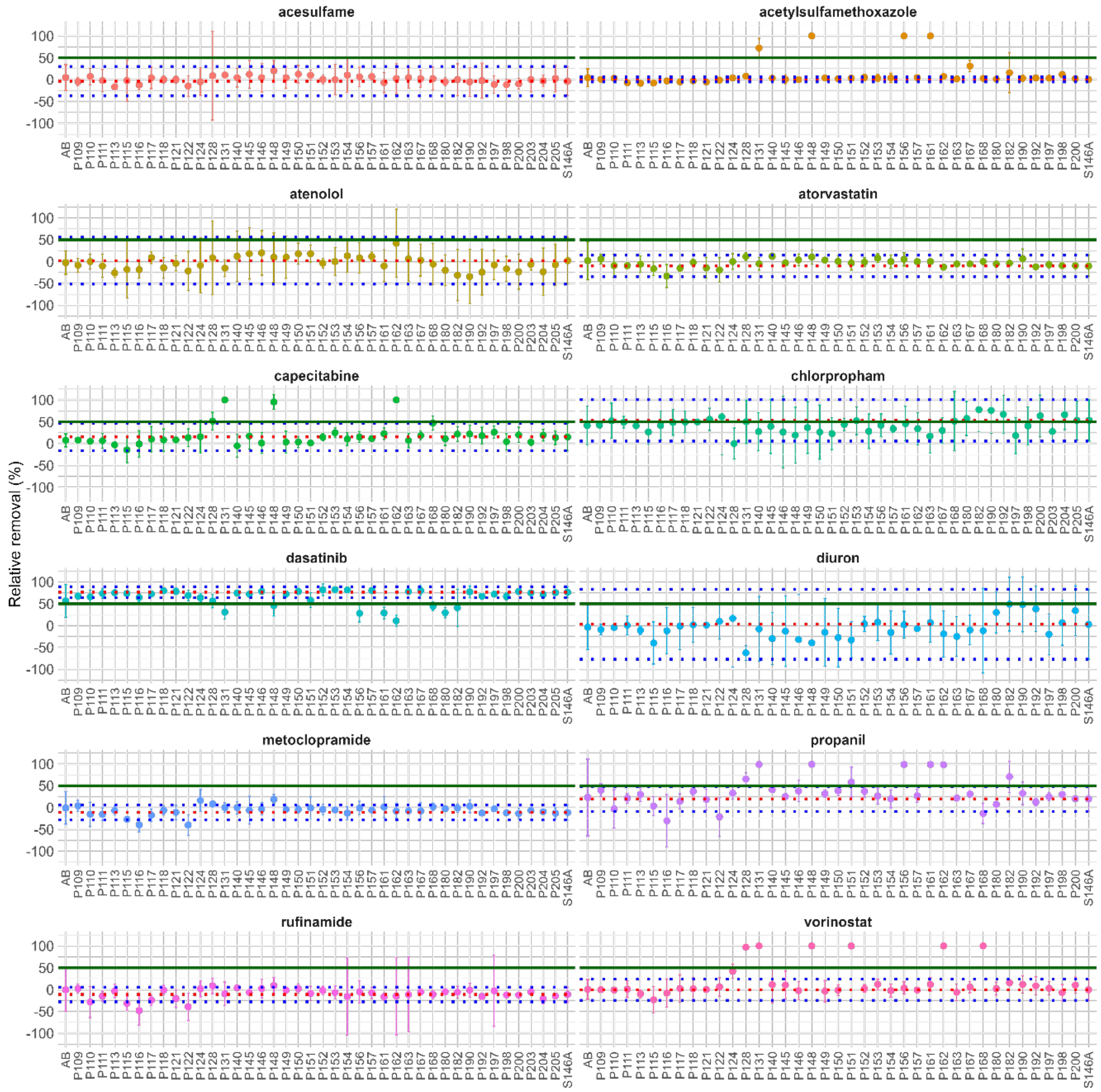

Figure S6: Relative removal of substrates by AS enzyme library. The green line marks the 50% removal threshold (first criterion for activity). AB represents the abiotic control, and S146A denotes the catalytically inactive variant P205-S146A. The dotted red line indicates the median of P205-S146A, while the blue lines represent the 1.5 IQR values of P205-S146A. Values above the media + 1.5 IQR of P205-S146a were considered to meet the second criterion for activity. These stringent criteria were selected to filter out false positives due to abiotic removal (e.g., chlorpropham, dasatinib). Different wavelengths were used for quantification: 225 nm (acesulfame, atenolol, chlorpropham, rufinamide), 252 nm (propanil, diuron, atorvastatin, acetylsulfamethoxazole, vorinostat), and 305 nm (dasatinib, metoclopramide, capecitabine). Data is from biological triplicates.

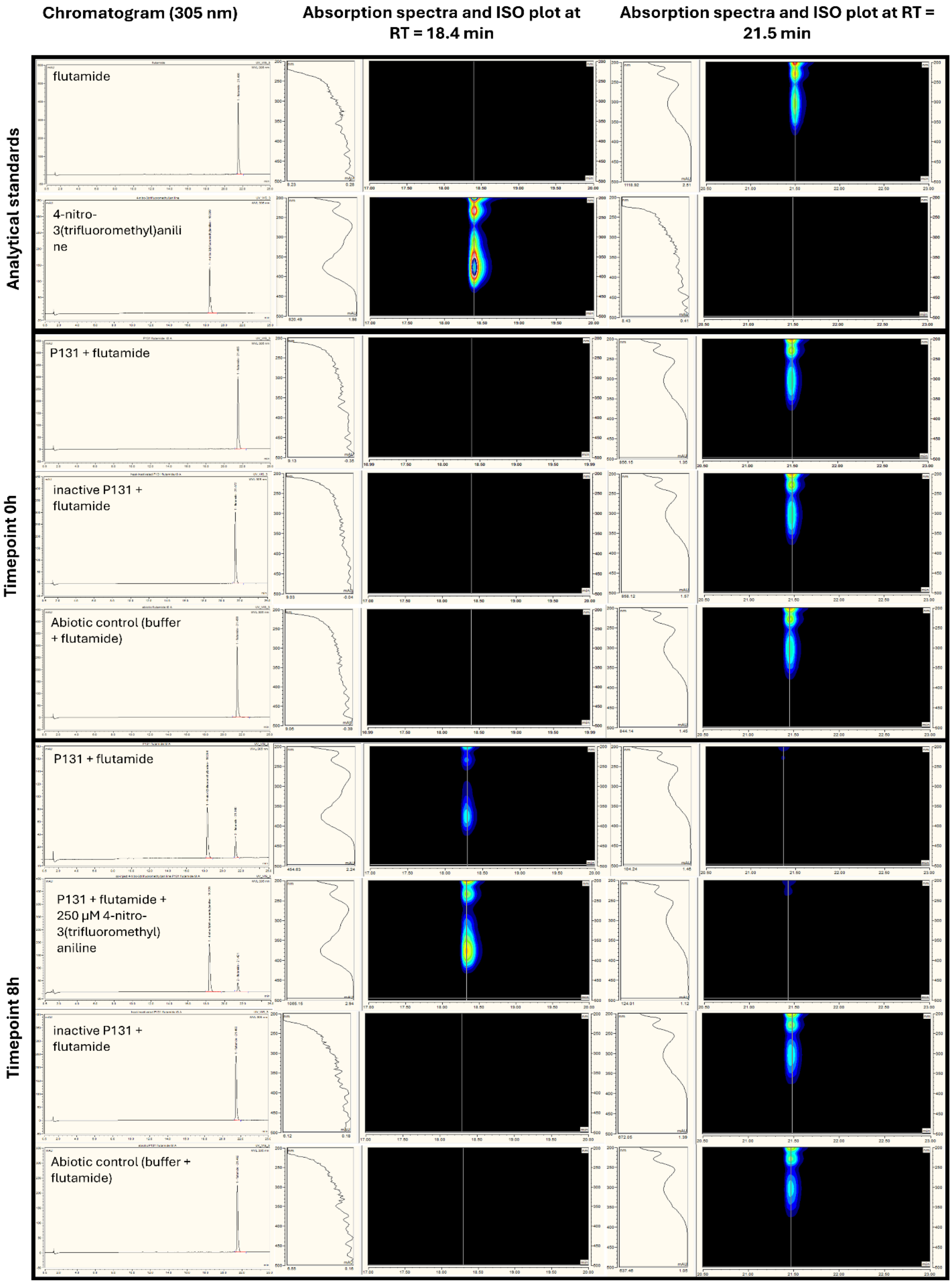
Chromatogram (305 nm)
Absorption spectra and ISO plot at RT = 18.4 min
Absorption spectra and ISO plot at RT = 21.5 min
Analytical standards
flutamide
4-nitro-3(trifluoromethyl)aniline
Timepoint 0h
P131 + flutamide
inactive P131 + flutamide
Abiotic control (buffer + flutamide)
Timepoint 8h
P131 + flutamide
P131 + flutamide + 250 µM 4-nitro-3(trifluoromethyl) aniline
inactive P131 + flutamide
Abiotic control (buffer + flutamide)

Figure S7: Flutamide transformation product verification. HPLC chromatograms at 305 nm, and 3D spectral profiles at retention times (RT) 14.8 min and 21.5 min. Top panels: analytical standards of flutamide and its amide-hydrolysis product, 4-nitro-3(trifluoromethyl)aniline. Middle panels: timepoint 0h samples from reactions containing flutamide with either purified P131, heat-inactivated P131, or an abiotic control. Bottom panels: timepoint 8h samples, including a co-injection of 250 µM 4-nitro-3(trifluoromethyl)aniline standard to the reaction with flutamide incubated with P131. The final P131 and heat-inactivated P131 concentrations were 0.04 mg/mL. All reactions were performed in 50 mM Tris-HCl pH 8 buffer.

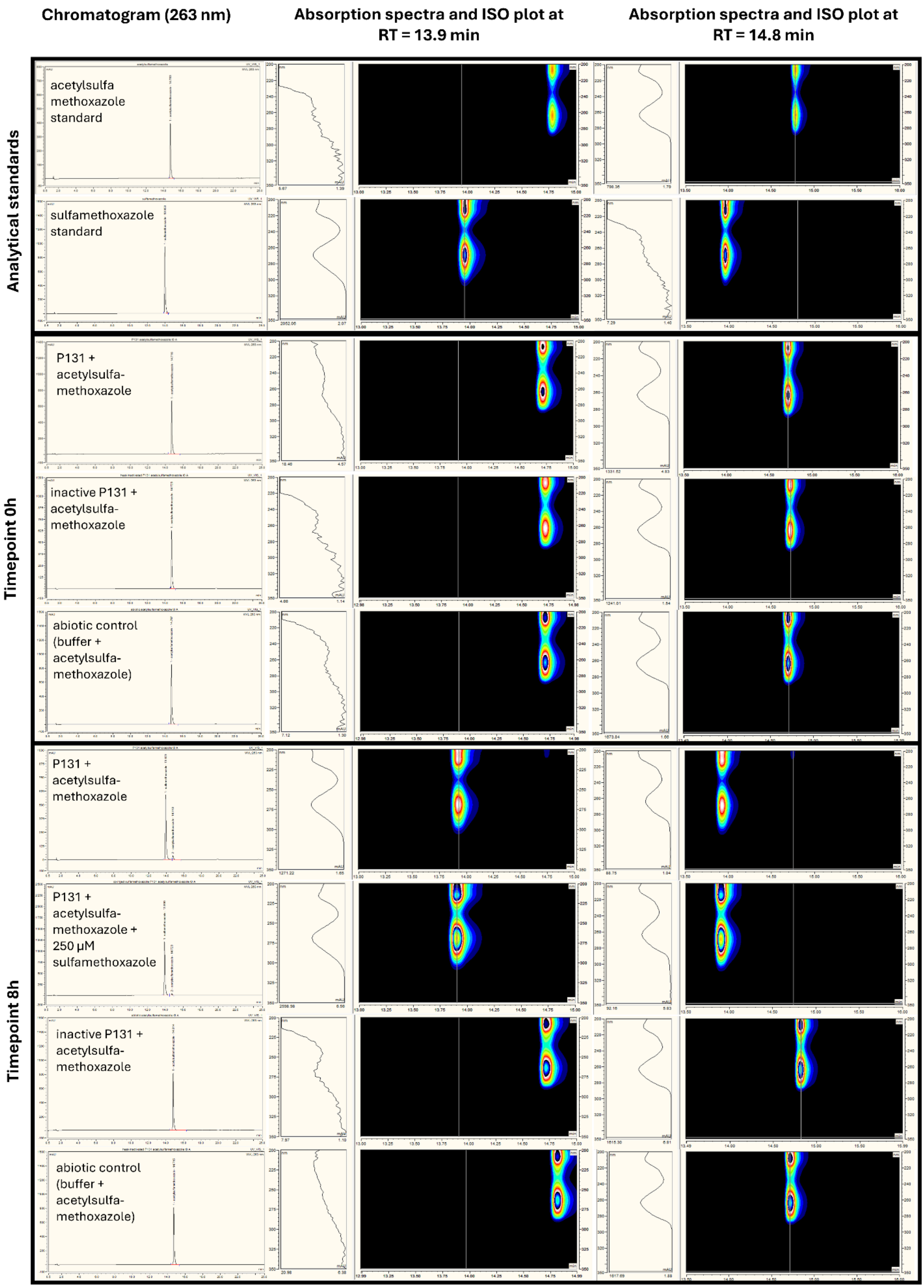

Chromatogram (263 nm)
Absorption spectra and ISO plot at RT = 13.9 min
Absorption spectra and ISO plot at RT = 14.8 min
Analytical standards
acetylsulfa methoxazole standard
sulfamethoxazole standard
Timepoint 0h
P131 + acetylsulfa-methoxazole
inactive P131 + acetylsulfa-methoxazole
abiotic control (buffer + acetylsulfa-methoxazole)
Timepoint 8h
P131 + acetylsulfa-methoxazole
P131 + acetylsulfa-methoxazole + 250 µM sulfamethoxazole
inactive P131 + acetylsulfa-methoxazole
abiotic control (buffer + acetylsulfa-methoxazole)

Figure S8: Acetylsulfamethoxazole transformation product verification. HPLC chromatograms at 263 nm, and 3D spectral profiles at retention times (RT) 13.9 min and 14.8 min. Top panels: analytical standards of acetylsulfamethoxazole and its amide-hydrolysis product, sulfamethoxazole. Middle panels: timepoint 0h samples from reactions containing acetylsulfamethoxazole with either purified P131, heat-inactivated P131, or an abiotic control. Bottom panels: timepoint 8h samples, including a co-injection of 250 µM sulfamethoxazole standard to the reaction with acetylsulfamethoxazole incubated with P131. The final P131 and heat-inactivated P131 concentrations were 0.04 mg/mL. All reactions were performed in 50 mM Tris-HCl pH 8 buffer.

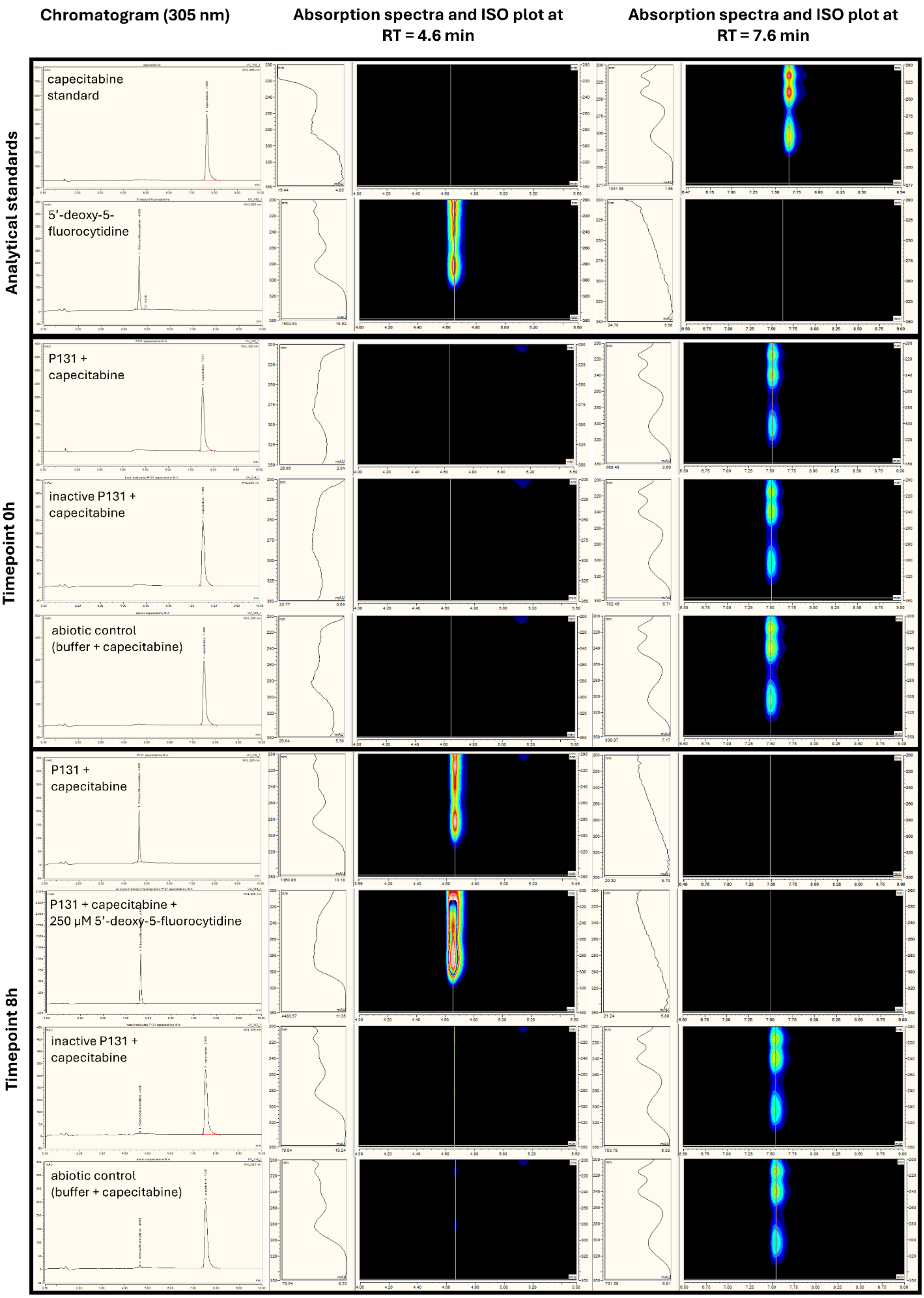
Chromatogram (305 nm)
Absorption spectra and ISO plot at RT = 4.6 min
Absorption spectra and ISO plot at RT = 7.6 min
Analytical standards
capecitabine standard
5'-deoxy-5-fluorocytidine
Timepoint 0h
P131 + capecitabine
inactive P131 + capecitabine
abiotic control (buffer + capecitabine)
Timepoint 8h
P131 + capecitabine
P131 + capecitabine + 250 µM 5'-deoxy-5-fluorocytidine
inactive P131 + capecitabine
abiotic control (buffer + capecitabine)

Figure S9: Capecitabine transformation product verification. HPLC chromatograms at 305 nm, and 3D spectral profiles at retention times (RT) 4.6 min and 7.6 min. Top panels: analytical standards of capecitabine and its amide-hydrolysis product, 5'-deoxy-5-fluorocytidine. Middle panels: timepoint 0h samples from reactions containing capecitabine with either purified P131, heat-inactivated P131, or an abiotic control. Bottom panels: timepoint 8h samples, including a co-injection of 250 µM 5'-deoxy-5-fluorocytidine standard to the reaction with capecitabine incubated with P131. The final P131 and heat-inactivated P131 concentrations were 0.04 mg/mL. All reactions were performed in 50 mM Tris-HCl pH 8 buffer.

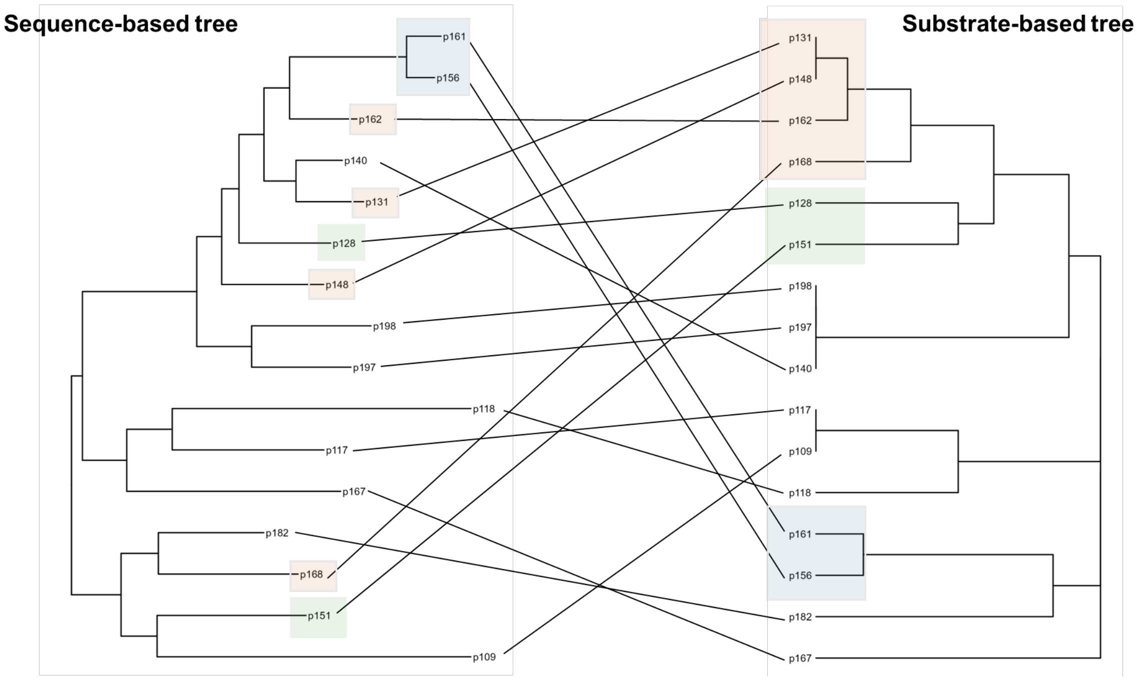

Figure S10: Substrate specificity dendrogram (excluding substrates that are not biotransformed by any enzyme) based on Jaccard index calculated from the substrate preference matrix. Colored boxes indicate the two clades/clusters with enzymes active with at least 4 substrates.

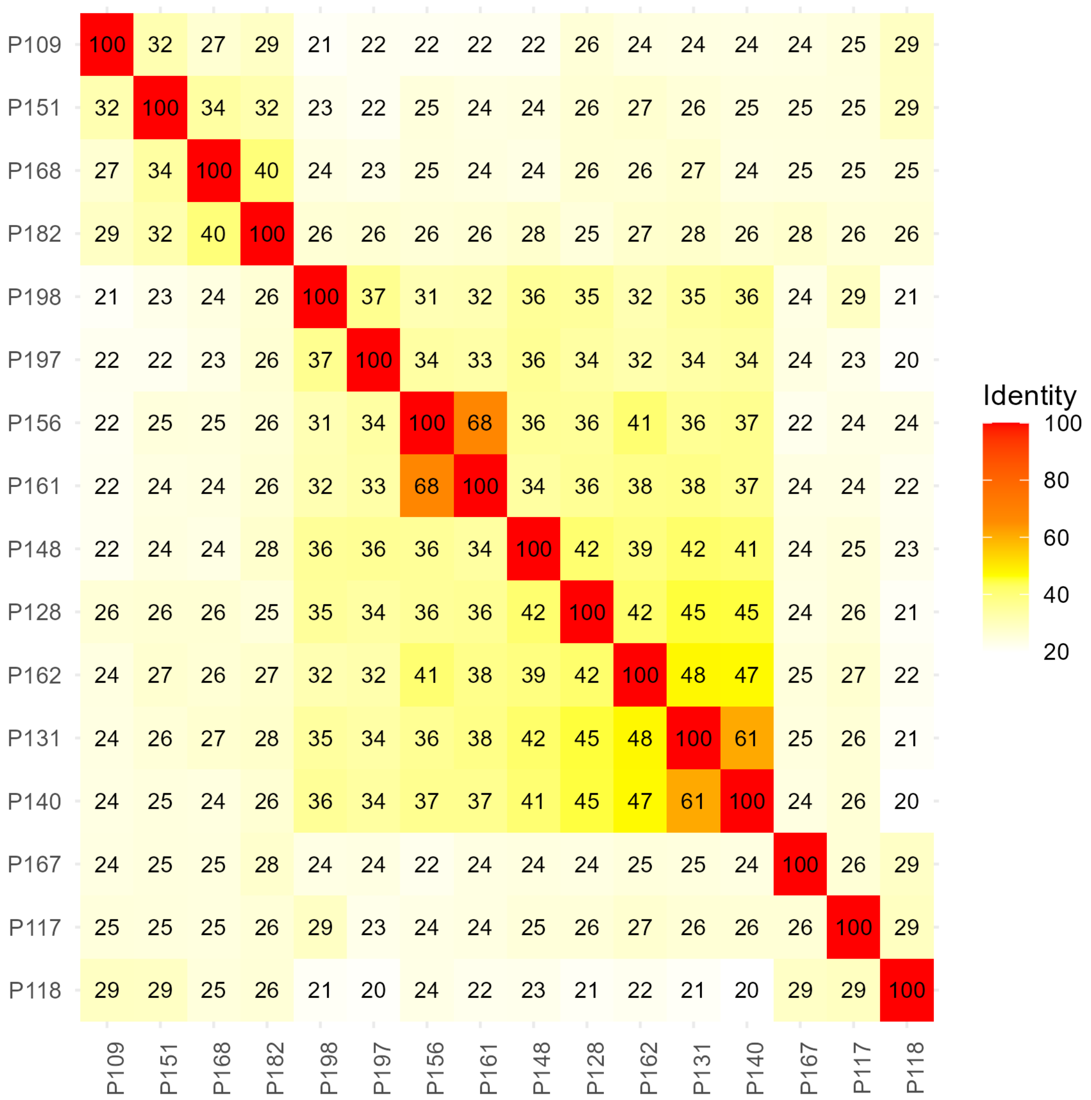

Figure S11: Protein identity matrix of the 16 enzymes which showed activity with at least one substrate.

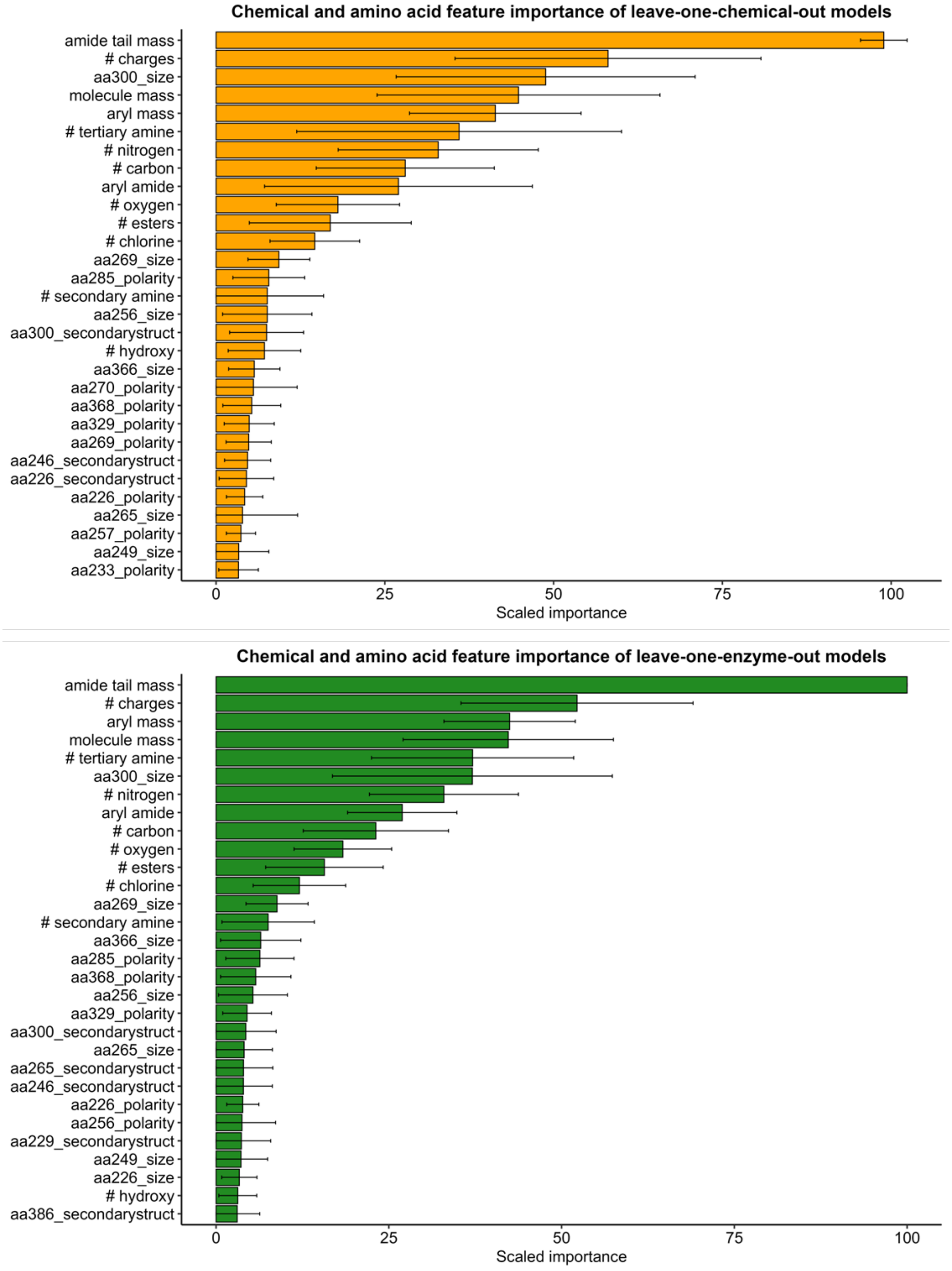

Figure S12: Top 30 chemical and amino acid scaled feature importance in XGBoost leave-one-chemical-out (top) and leave-one-enzyme-out (bottom) models. Amino acid numbers refer to positions in the protein alignment of 16 AS enzyme hits.

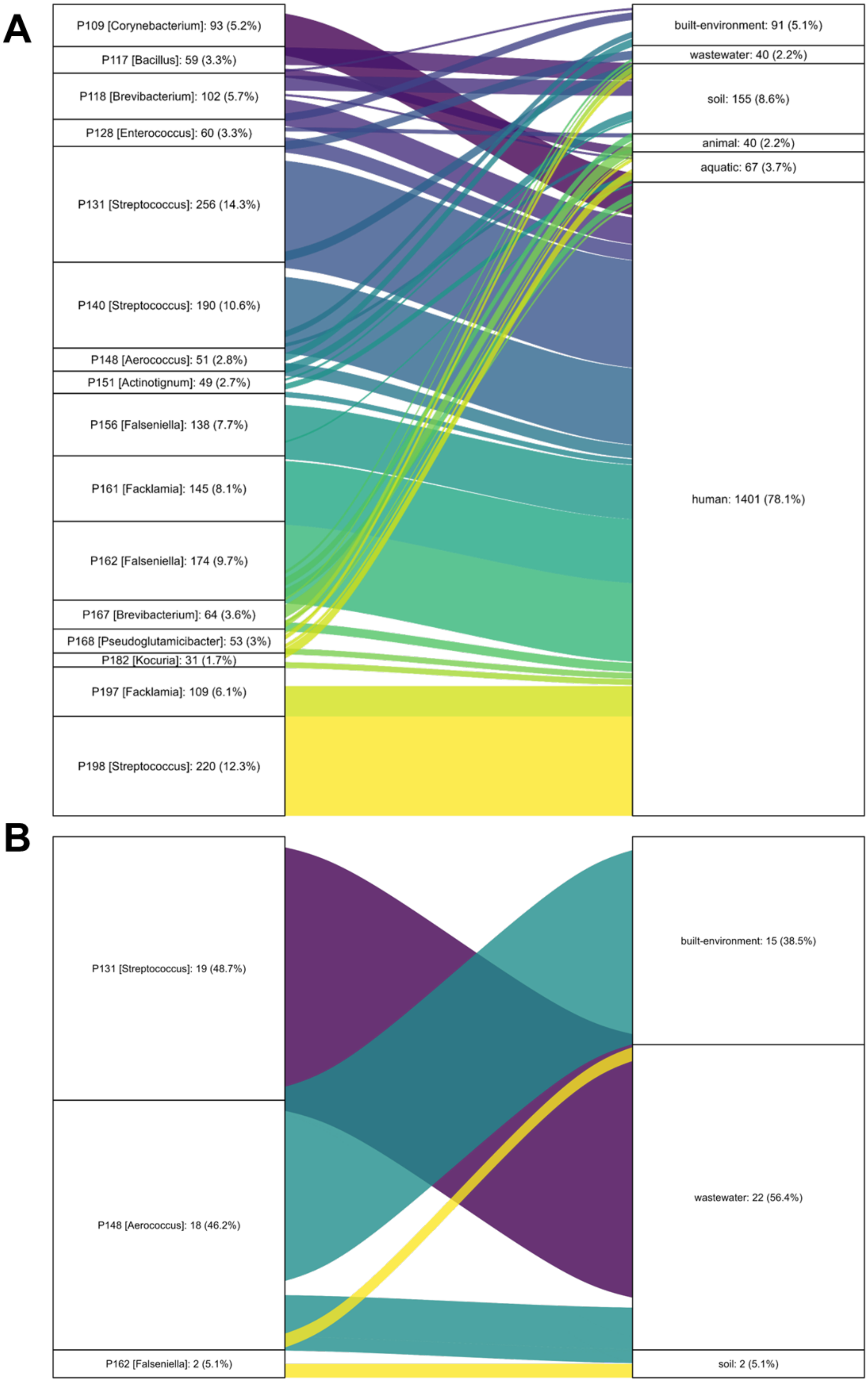
A
P109 [Corynebacterium]: 93 (5.2%)
P117 [Bacillus]: 59 (3.3%)
P118 [Brevibacterium]: 102 (5.7%)
P128 [Enterococcus]: 60 (3.3%)
P131 [Streptococcus]: 256 (14.3%)
P140 [Streptococcus]: 190 (10.6%)
P148 [Aerococcus]: 51 (2.8%)
P151 [Actinotignum]: 49 (2.7%)
P156 [Falseniella]: 138 (7.7%)
P161 [Facklamia]: 145 (8.1%)
P162 [Falseniella]: 174 (9.7%)
P167 [Brevibacterium]: 64 (3.6%)
P168 [Pseudoglutamicibacter]: 53 (3%)
P182 [Kocuria]: 31 (1.7%)
P197 [Facklamia]: 109 (6.1%)
P198 [Streptococcus]: 220 (12.3%)
built-environment: 91 (5.1%)
wastewater: 40 (2.2%)
soil: 155 (8.6%)
animal: 40 (2.2%)
aquatic: 67 (3.7%)
human: 1401 (78.1%)
B
P131 [Streptococcus]: 19 (48.7%)
P148 [Aerococcus]: 18 (46.2%)
P162 [Falseniella]: 2 (5.1%)
built-environment: 15 (38.5%)
wastewater: 22 (56.4%)
soil: 2 (5.1%)

Figure S13: Distribution of enzyme homologs across different environments (according to the Global Microbial Gene Catalog). **A**) Prevalence of active enzyme homologs in environmental, animal and human microbiomes. **B**) Prevalence of the most promiscuous enzymes in the study in environmental microbiomes. Each stratum represents an enzyme, with the bacterial host genus indicated in brackets. The connections between strata represent the prevalence of each enzyme in various environments. The labels on the strata display both the absolute counts and the relative percentages of the enzyme homologs.

References

(1) Yu, Y.; Trottmann, N. F.; Schärer, M. R.; Fenner, K.; Robinson, S. L. Substrate Promiscuity of Xenobiotic-Transforming Hydrolases from Stream Biofilms Impacted by Treated Wastewater. *Water Res.* **2024**, *256*, 121593.
